# Low Frequency of Human Papillomavirus in Strictly Site-Coded Oral Squamous Cell Carcinomas, Using the Latest NHI/SEER-ICD Systems: A Pilot Observational Study and Critical Review

**DOI:** 10.3390/cancers13184595

**Published:** 2021-09-13

**Authors:** Vera Panzarella, Giuseppina Campisi, Ylenia Giardina, Laura Maniscalco, Giuseppina Capra, Vito Rodolico, Olga Di Fede, Rodolfo Mauceri

**Affiliations:** 1 Department of Surgical, Oncological and Oral Sciences (Di.Chir.On.S), University of Palermo, 90127 Palermo, Italy; vera.panzarella@unipa.it (V.P.); ylenia.giardina@community.unipa.it (Y.G.); olga.difede@unipa.it (O.D.F.); rodolfo.mauceri@unipa.it (R.M.); 2 Department of Biomedicine, Neuroscience and Advanced Diagnostics (BIND), University of Palermo, 90127 Palermo, Italy; maniscalco.laura92@gmail.com; 3 Department of Health Promotion, Mother and Child Care, Internal Medicine and Medical Specialties (ProMISE), University of Palermo, 90127 Palermo, Italy; giuseppina.capra@unipa.it (G.C.); vito.rodolico@unipa.it (V.R.); 4 Department of Biomedical and Dental Sciences, Morphological and Functional Images, University of Messina, 98124 Messina, Italy; 5 Department of Dental Surgery, Faculty of Dental Surgery, University of Malta, 2090 Msida, Malta

**Keywords:** human papillomavirus, oral squamous cell carcinoma, NIH/SEER ICD-0-3.2, PCR HPV-DNA, p16 IHC

## Abstract

**Simple Summary:**

The incidence of oropharyngeal squamous cell carcinomas (OPSCCs) has increased in the last decades, and this seems to be correlated to the infectious epidemiological trend of human papillomavirus (HPV). The prevalence of HPV-positive OPSCCs is approximately 70%, with involvement mainly of the tonsillar area. On the role of HPV in oral squamous cell carcinoma (OSCC), very few studies have investigated the prevalence of HPV in strictly site-codified OSCC, excluding the base of the tongue as distinct oropharyngeal entity. As a result, an inappropriate estimation of HPV infection in OSCC has been observed. We investigated HPV status, using a combination of detection methods, in a sample of 40 subjects with OSCC coded by the latest site classifications. Moreover, we performed a critical review of the studies with the same outcomes. The main finding of our investigation was a low frequency of HPV-positive OSCC, suggesting no significant HPV role in strictly oral carcinogenesis.

**Abstract:**

The aim of this study was to evaluate HPV status in oral squamous cell carcinoma (OSCC), as coded by the latest classifications and applying a combination of detection methods used in clinical practice. Forty-two patients with suspect OSCC were consecutively recruited. Patients underwent an incisional biopsy for histological OSCC diagnosis and HPV identification by PCR DNA and p16 IHC. All lesions were coded by the latest ICD-0-3.2 site/histology classifications, as proposed for OSCC by the National Cancer Institute Surveillance, Epidemiology and End Results Programs. Moreover, a comparative analysis review, critically evaluated by the same site-coded systems and HPV detection methods, was performed. In 40 confirmed cases of OSCC, the frequency of HPV infection was 10% (4/40). Among positive patients, two cases were PCR DNA/p16 IHC positive (high-risk HPV 51, high-risk HPV 67), two cases were PCR DNA positive/p16 IHC negative (high-risk HPV 31 + 68, high-risk HPV 66). Applying the latest site coding systems for OSCC, the frequency of HPV infection in this study and in similar, reviewed investigations was low (from 3.3% to 12.5%). These results suggested no significant HPV role in oral carcinogenesis, particularly where an updated site-coded classification of OSCCs (categorically excluding the base of the tongue) had been performed.

## 1. Introduction

The incidence of oropharyngeal squamous cell carcinomas (OPSCCs) has increased considerably in the last four decades. This trend appears to be related to an incidence of human papillomavirus (HPV) infection: indeed, the prevalence of HPV-positive OPSCCs is approximately 70% (60% supported by HPV16/18 genotypes, and 10% supported by HPV31/33/45/52/58 genotypes) [1]. HPV-related OPSCCs appear to occur predominantly on the tonsillar area and on the base of tongue, while HPV-negative OPSCCs, which are linked to traditional risk factors, tend to involve other oral and oropharyngeal sub-sites.

HPV-positive OPSCCs seem to have a better prognosis than HPV-negative OPSCCs due to a lower risk of local recurrence and an increased radio-chemo sensitivity [2,3]. Consequently, the eighth edition of the TNM staging system, related to HPV-positive OPSCCs, has been updated to reflect the varying prognoses of HPV-related head and neck cancers more accurately [4,5]. However, HPV detection rates still present a wide variation in OPSCCs, which is due to two compellingly interconnected reasons: (i) a non-univocal topographical classification of the OPSCC; and (ii) the different and various HPV identification techniques used [6,7].

These issues become even more critical if the primary outcome is the prevalence of HPV in strictly oral cavity squamous cell carcinomas (OSCCs). This is in part due to the limited number of studies with detailed coding of intra-oral sites, thereby distinguishing the latter from oropharyngeal sites (especially the base of the tongue).

Since 2013 to the current day, the International Classification of Disease for Oncology (ICD-0) has revised the topographical classification of oral and oropharyngeal sites by classifying the oral cavity sites within a single group, which is now coded from C02.0 to C06.9 [8]. However, very few studies regarding the prevalence of HPV in OSCCs have drawn a correct site-codified distinction of oral sites, particularly of tongue sites, and most of the investigations repeat the error of using the generic term NOS (not otherwise specified) ‘tongue (C02.9)’ and/or ‘mouth (C06.9)’, without distinguishing the base of tongue as a distinct oropharyngeal, morphological entity. Thus, an overestimation of the HPV infection data in OSCC has been observed [9]. For these reasons, the National Cancer Institute Surveillance, Epidemiology, and End Results Programs (NIH/SEER) have updated the head and neck terminology and definitions relating to the oral cavity and mobile tongue, definitively confirming the inclusion of the base of the tongue (C01.9) among the oropharyngeal sites under consideration [10]. This update constitutes an explicit invitation to apply this distinction to future studies of oral carcinogenesis, especially with respect to the HPV status.

With reference to HPV detection techniques, while the gold standard to demonstrate viral oncogenic activity remains the choice for detection of HPV E6 and E7 messenger RNA (mRNA) expression via a quantitative reverse transcription polymerase chain reaction (qRT-PCR), in clinical practice it is considered easier to use the tumor suppressor protein encoded by CDKN2A gene (9p21.3) (p16) immunohistochemistry (IHC). The p16 tumor suppressor protein (INK4A) regulates retinoblastoma protein (pRb) and tumor suppressor protein encoded by suppressor gene TP53 (17p13.1) (p53) pathways. A loss of pRB function following E7 protein HPV activity leads to increased p16 overexpression [11]. Consequently, it is considered a reliable surrogate marker of the transcriptional activity of high risk (hr) HPV infection. Conversely, some authors have reported up to 20% of p16-positive OPSCCs as HPV-negative, in the absence of an etiological role for HPV in the carcinogenic process. Furthermore, several other studies have demonstrated that an HPV status, only defined by p16 IHC, may be an insufficiently specific procedure [12,13]. Thus, a polymerase chain reaction (PCR) DNA is often combined with p16 IHC to confirm the presence of HPV-DNA due to its known advantages (i.e., low cost, heightened sensitivity and wide availability in clinical/surgical settings and laboratories).

Bearing in mind these considerations, the main aim of this paper was to conduct an observational pilot study to assess the frequency of HPV in a collection of OSCCs, applying the newest ICD-0-3.2 site/histology classification, as proposed by NIH/SEER for oral cavity and mobile tongue [10]. A combination of HPV identification techniques (p16 IHC and PCR HPV-DNA), which are usually available in clinical practice, was deployed. Moreover, a critical review of similar investigations was performed in order to perform a comparative analysis between the results obtained, having applied the same site-coded OSCCs classification systems and HPV detection and sampling methods.

## 2. Materials and Methods

### 2.1. Observational Study

The study protocol conformed to the ethical guidelines of the 1964 Declaration of Helsinki and its later amendments or comparable ethical standards. It was also approved by the institutional review board of the “Paolo Giaccone” Policlinico University Hospital in Palermo (Italy) (approval number 11/2011). All patients signed written informed consent prior to specimen collection.

#### 2.1.1. Entry Criteria

Patient recruitment commenced on 1 May 2018 and finished in December 2020. All participants were consecutively recruited from the Unit of Oral Medicine at the “Paolo Giaccone” Policlinico University Hospital in Palermo (Italy).

The eligibility criteria were:

(i). age ≥ 18 years;
(ii). ability to provide informed consent;
(iii). suspected OSCC, located strictly in the oral cavity, having applied the 2021 NIH/SEER ICD-0-3.2 topographical classification codes (from C02.0 to C02.3 for the tongue, C03.0 and C03.1 for the gum, C04.0 and C04.1 for the floor of the mouth, C05.0 and C05.1 for palate, and C06.0, C06.1 and C06.2 for cheek mucosa, mouth vestibule and retromolar area); the codes referring to not otherwise specified (NOS) oral sites have not been considered [8,10];
(iv). no previous diagnosis of cancer in the head or neck regions.

#### 2.1.2. Data Collection and Clinical Examination

Forty-two patients with highly suspicious OSCC were interviewed, using a structured, pre-tested baseline questionnaire. During the interview, variables including socio-demographic data, medical history, and a previous diagnosis of cancer were recorded. To assess health-related variables, the patients were interviewed as to their current and lifetime smoking history, alcohol consumption, and frequency of dental attendance. Regarding tobacco use, patients were classified as ‘never’, ‘current’, or ‘former’ smokers (if they had quit smoking at least 1 year prior to the study). Smokers were classified into three categories according to their cumulative tobacco consumption: (a) 0 (non-smokers), (b) <25 packs per year (light-smokers), and (c) ≥25 packs per year (moderate/heavy-smokers). Alcohol consumption was defined in terms of drink units (DU) per week: (a) non-drinkers (who had never consumed alcohol or who had less than one drink per week); moderate-drinkers (<16 DU-week), and heavy-drinkers (≥16 DU-week).

Each lesion was classified according to the latest 2021 NIH/SEER ICD-0-3.2 topographical classification codes, as reported in the eligibility criteria above; any potential local risk factors (mechanical trauma, such as sharp cusps, and incongruous prosthesis) were discriminated and recorded. The details of the respective site/codes of overlapping lesions were annotated.

#### 2.1.3. Sample Collection

To confirm a clinical diagnosis, a histopathological examination was undertaken for all patients. After local anesthesia, an incisional biopsy was performed with a scalpel punch. Specimens were obtained from each patient from the same non-necrotic area of the suspected carcinoma. The section from one sample was fixed in formalin solution and sent to the pathology laboratory for histopathological SCC diagnosis and p16 IHC examination; a second section from a fresh sample was sent to the microbiology laboratory for the PCR HPV-DNA test.

#### 2.1.4. Histological Examination and p16 Immunohistochemistry

A microscopic evaluation was performed by an oral pathologist. Formalin-fixed, paraffin-embedded (FFPE) sections of 5 µm were stained with routine hematoxylin and eosin, and examined to confirm a diagnosis of OSCC. Thereafter, the degree of differentiation, according to the WHO grading system, was determined. Only oral SCCs, coded as 807*/* by an ICD-0-3 SEER site/histology validation list, were considered. Paraffin-embedded tissue sections of 4 µm were used for IHC staining, using the CDKN2A/p16INK4a antibodies (Ventana Ref 805–4713, pre-diluted ready to use, clone E6H4). Subsequent stages were performed with the ultraView universal 3,3′-diaminobenzidine tetrahydrochloride (DAB) detection kit (Ventana). A Ventana Benchmark XT auto stainer (Ventana Medical Systems, Inc., Tucson, AZ, USA) was used for immunohistochemical studies. p16 staining intensity was scored as: 0 = negative (<50% diffuse and strong nuclear and cytoplasmic staining), 1 = equivocal (<70% but >50% diffuse and strong nuclear and cytoplasmic staining), and 2 = positive (>70% diffuse and strong nuclear and cytoplasmic staining), according to the “Template for reporting results of biomarker testing of specimens from patients with tumors of the head and neck” from the College of American Pathologists (Version: Head Neck Biomarkers 1.0.0.0; https://tinyurl.com/y2u9m8b6 (accessed on 31 December 2020)).

#### 2.1.5. DNA Extraction and HPV DNA Detection

To isolate viral DNA, two fresh sample sections of 5 μm were incubated overnight at 56 °C in 250 μL of a 0.5% Tween 20, 50 mM Tris–HCl pH 8.5, 1 mM EDTA, containing 300 μg/mL of proteinase K solution. Proteinase K was inactivated at 95 °C for 10 min. The samples were centrifuged for 5 min at 13,000 rpm, and total DNA was extracted with a High Pure PCR Template Preparation kit (Roche Diagnostics GmbH, Mannheim, Germany), following the manufacturer’s instructions.

Histologically confirmed OSCCs were checked for DNA quality by the standard amplification of human beta-globin; only beta-globin-positive samples were examined for HPV DNA. The samples were tested in duplicate, and the amplification controls were blank. The HPV DNA negative human Wi-38 cell line was used as a negative control and the HPV DNA positive human cell line SiHa (1–2 copies of HPV-16 DNA per cell) was used as a positive control. SiHa dilutions from 104 (10,000–20,000 HPV-16 DNA copies) down to 10–1 cell (1–2 copies) were used to check amplification sensitivity. Amplifications were carried out in a DNA thermal cycler (Mastercycler, Eppendorf, Hamburg, Germany) and the PCR products were analyzed in 8% polyacrylamide gel.

The presence of HPV DNA was detected using two HPV assays. The INNO-LiPA HPV Genotyping Extra II kit (Fujirebio Diagnostics, Inc, Great Valley Parkway, Malven, PA, USA), based on the combined use of SPF10 PCR and LiPA hybridization, was employed. The SPF general primers detected at least 43 different HPV genotypes, and the LiPA type-specific assay identified 32 types: 20 hrHPV (HPV16, HPV18, HPV26, HPV31, HPV33, HPV35, HPV39, HPV45, HPV51, HPV52, HPV53, HPV56, HPV58, HPV59, HPV68, HPV66, HPV67, HPV70, HPV73 and HPV82) and 12 low-risk HPV (lrHPV): HPV6, HPV11, HPV40, HPV42, HPV43, HPV44, HPV54, HPV61, HPV62 HPV81, HPV83 and HPV89). Due to the higher number of HPV types detected by the SPF10 primers than the LiPA assay, various samples yielded SPF10-positive/LiPA-negative results. These HPV types were subsequently amplified by a highly sensitive nested PCR assay, consisting of a first step of amplification with the PGMY09/11 primer pair, followed by a second step with the GP05+/GP06+ primers. The HPV genotyping procedure was based on the direct sequencing of PGMY/GP-PCR fragments, utilizing consensus nested primers as sequencing primers. In brief, the amplification products were purified by Microcon1 YM-100 Filter Devices (Amicon; Millipore, Billerica, MA, USA), and approximately 5 μg of product was added to 4 μg of BigDyeTM Terminator Ready Reaction mix (Applied Biosystems, Foster City, CA, USA). The purification of reaction mixtures and removal of free BigDyeTM was performed by Centrisep Spin Columns (Princeton Separations, Adelphia, NJ, USA), and the mixture was analyzed on an ABI PRISM1 310 Genetic Analyzer (Applied Biosystems). Alignments were obtained from the online BLAST server. HPV types were classified as low risk (LR) or hrHPVs, according to a more recent classification (online HPV Database http://www.hpv-web.lanl.gov (accessed on 31 December 2020)).

### 2.2. Critical Review

#### 2.2.1. Focused Questions

▪ How many studies, published in the last decade and regarding the frequency of HPV in OSCC, report a standardized coding of oral sites?
▪ Are there differences in the frequency of HPV status when the ‘not otherwise specified (NOS) tongue’ are excluded?
▪ Are there discordant results regarding the frequency of HPV between PCR DNA (considered the gold standard) and p16 IHC?

#### 2.2.2. Search Criteria

Of the studies relating to the frequency of HPV in OSCC, only those investigating the status of HPV from tissue specimens (FF = formalin-fixed; PE = paraffin embedded; FFPE= formalin fixed/paraffin embedded) of strictly OSCCs were examined. Moreover, studies omitting a well-defined site classification for oral cavity were excluded, in addition to studies not using a combination of HPV identification techniques (PCR DNA and p16 IHC). Finally, only those scientific studies in which OSCC had been confirmed by histological investigation were selected. Studies were identified by an electronic search of scientific articles from different biomedical databases (i.e., PubMed, Ovide/MEDLINE, Web of Knowledge, Embase) and by scanning reference lists of articles. Only studies published in English from 2010 to 2020 were considered eligible. The following search terms, used separately and jointly, were deployed: oral squamous cell carcinoma, oral carcinoma, oral cancer, OSCC, papillomaviridae, human papilloma virus, HPV, human papillomavirus, polymerase chain reaction, PCR, and p16 IHC. The eligibility assessment was performed independently by two reviewers (VP and RM); any difference of opinion between the reviewers was resolved by consensus. The studies were initially selected by applying the inclusion and exclusion criteria of the title and the abstract. Duplicate papers were removed, and selected articles were scrutinized to assess eligibility.

#### 2.2.3. Comparison Data Criteria

To guarantee an optimally fit data comparison, a re-coding of the oral sites, applying the same site-coding models used by the authors of this paper, was conducted for each study. In cases in which no details relating to tongue sites (dorsal surface of tongue/C02.0, border of tongue/C02.1, ventral surface of tongue/C02.2 and anterior 2/3 of tongue/C02.3) were identified, the generic code ‘tongue NOS’ (C02.9) was used.

### 2.3. Statistical Analysis

Statistical units were defined as ‘patients who satisfied the inclusion criteria of the study’. Continuous variables were summarized with mean values and standard deviations, while categorical variables were expressed as counts and percentages. Confidence intervals at the 95% confidence level (95% CI) for proportions were computed using the conservative Clopper–Pearson method. Moreover, pairwise comparisons between pairs of proportions with a correction for multiple testing using Holm as the *p*-value adjustment method were calculated. With reference to the sample described in this observational study and those of each reviewed study, the sensitivity and specificity of the p16-IHC technique, compared to PCR HPV-DNA (considered as the gold standard), were calculated, and expressed as percentages and 95% exact confidence intervals. Statistical analysis was performed with R software (version 4.0.2).

## 3. Results

### 3.1. Observational Study

Of the 42 patients with suspicious OSCCs, 40 (No. 40) patients with histologically confirmed OSCC were included. All demographic and clinical data have been collated in Table 1.

Regarding sex and habits, most of the patients were males (23/40, 57.5%), with a mean age of 66.5 ± 14.1 years (range 30–91 years). The HPV-positive patients were all male, with a mean age of 58.7 ± 8.7 years (range 44–64 years), while the HPV-negative patients had a mean age of 67.4 ± 14.3 years (range 30–91 years). Eighteen patients were moderate-heavy smokers (45%), and three patients were light smokers (7.5%), while three patients were classified as heavy drinkers (7.5%). Regarding the occurrence of other potential risk factors, a clinical examination revealed the presence of mechanical trauma in 15 patients, including: sharp cusps in 12 patients (30%) and incongruous prostheses in three patients (7.5%). Only 13 patients (32.5%) had no harmful habits or mechanical trauma (Table 1).

With reference to OSCC sites, the border of tongue (C02.1) was the most commonly affected site (13/40; 32.5%, 95% CI = [19–49%]), followed by the buccal mucosa (C06.0; 7/40; 17.5%, 95% CI = [7–33%]), the retromolar area (C06.2; 7/40; 17.5%, 95% CI = [7–33%]), the anterior floor of mouth (C04.0; 4/40; 10%, 95% CI = [3–24%]), overlapping tongue lesions (C02.8= C02.1 + C02.2; 4/40; 10%, 95% CI = [3–24%]), the hard palate (C05.0; 3/40; 7.5%, 95% CI = [2–20%]), and the gums (C03.1; 2/40; 5%, 95% CI = [0.6–17%]) (Table 2).

**Figure 1:**
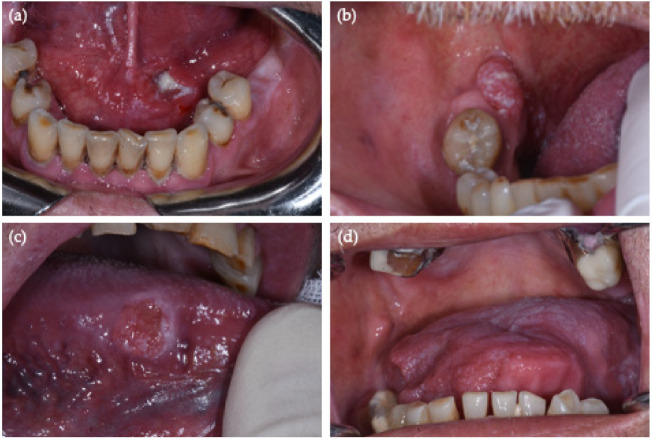
HPV-positive OSCCs: (**a**) anterior oral floor (C04.0—hrHPV 51) and (**b**) retromolar area (C06.2 hrHPV 67) were p16 positive—PCR HPV-DNA positive; (**c**) border of the tongue (C02.1—hrHPV 31 + 68) and (**d**) border of the tongue (C02.1—hrHPV 66) were PCR HPV-DNA positive and p16 IHC negative.

Only four OSCC samples were HPV-DNA PCR positive (4/40; 10%, 95% CI = (3–24%)), of which two occurred on the border of the tongue (C02.1), one on the anterior floor of the mouth (C04.0) and one in the retromolar area (C06.2) (Figure 1a–d, Table 3). Those HPV-positive patients were all male smokers, who presented with non-keratinized mucosa.

Two cases of OSCC (one anterior floor of the mouth, C04.0, and one retromolar area, C06.2) were both PCR HPV-DNA and p16 IHC positive (5%, 95% CI = [0.6–17%]) with hrHPV 51 and hrHPV 67 genotypes, respectively. The two cases of OSCC on the border of the tongue (C02.1) were PCR HPV-DNA positive and p16 IHC negative (5%, 95% CI = [0.6–17%]); one was positive for the hrHPV 31 + 68 genotypes and the hrHPV 66 genotype (PCR HPV-DNA positive), respectively. The study sample showed a sensitivity regarding the p16-IHC technique, compared to PCR HPV-DNA, which was equal to 50% (2/4, 95% CI = [6–93%]) in combination with a specificity of 100% (36/36, 95% CI = [90–100%]) (data not shown). It was calculated that this sample size is sufficient to estimate 10% HPV-positive cases in OSCC assuming 95% as confidence level and 9% as error margin. The sample size calculation showed that, with 40 patients and an estimate of 10% of HPV-positive cases in OSCC, it could be possible our estimate will diverge from the true value of the parameter not more than 9% in absolute value, compared to the usual 5%.

### 3.2. Critical Review

Of a total of 61 studies potentially eligible to satisfy the study criteria and for which a search was made during the 2010–2020 period, 13 were selected and critically reviewed [14,15,16,17,18,19,20,21,22,23,24,25,26]. A list of the studies, with the 2021 NIH/SEER ICD-0-3.2 site-coded classification and the frequency results of HPV status with PCR DNA and with p16-IHC, is reported in Table 4. The overall HPV frequency, obtained from PCR DNA, ranged from 0 to 48%. Only three studies reported a distinction between the ‘anterior 2/3 of tongue/C02.3′ and the generic ‘tongue, NOS (C02.9)’, with the following HPV frequency rates: Laco et al. 3/24 (12.5%, 95% CI = [2–32%]), Emmet et al. 5/63 (8%, 95% CI = [3–18%]), and Vidal Loustao et al. 5/152 (3.3%, 95% CI = [1–7%]) [15,17,18]. Of these three studies, only two [17,18] reported data relating to a p16 investigation, with p16 IHC positive results only regarding one case out of five PCR HPV-DNA positive cases. The adjusted pairwise comparisons among HPV frequencies in the sample described in this paper and those from the three studies by Laco et al., Emmet et al. and Vidal Loustao et al. revealed no statistically significant differences in the percentage of positive HPV, both by PCR DNA and p16 (adjusted *p*-value > 0.05). Referring to detection techniques, only Duncan et al. identified all the HPV-positive cases with both PCR DNA and P16-IHC techniques (100%, 95% CI = [59–100%], with a specificity of 90.6%, 95% CI = [45–67%]) [21]. The other analyzed studies showed a low combination of sensitivity and specificity of the P16-IHC technique compared to PCR HPV-DNA.

## 4. Discussion

In the last 50 years, the role of HPV infection in oral carcinogenesis has been extensively investigated. The wide variation in detection rates of oral HPV in potentially/overtly malignant lesions (0–100%), can be explained by two variables: (i) the extreme heterogeneity of the HPV detection techniques; and (ii) the absence of a standardized classification of the sampling site [4,7,27]. Referring to the latter, it is important to underline that in a high percentage of the studies regarding HPV and OSCCs conducted in the last 20 years, the precise anatomic site of the samples (oral vs oropharyngeal) was not provided, and thus the effective impact of HPV infection in strictly oral carcinogenesis could not be determined.

Consequently, to assess the effective prevalence of HPV infection in the oral cavity, and especially in OSCCs, it is essential to perform standardized diagnostic procedures and to apply an unequivocal site-coding system.

The aim of our observational study was to evaluate the frequency of HPV infection in a collection of 40 SCCs from strictly oral cavity sites, as codified by the 2021 NIH/SEER ICD-0-3.2 site/histological classification systems [8,10]. This evaluation was made with reference to a combination of HPV tests, from those most commonly available in clinical practice (p16 IHC and PCR HPV-DNA). Specifically, two-step diagnostic algorithms were performed: PCR for HPV DNA, to viral detection and genotyping (high specificity), followed by p16 IHC to investigate the viral transcriptional activity (high sensitivity), as suggested by Qureishi et al. [28]. To eliminate a potential selection bias related to different/non-site coded sampling, histological specimens from the same sample site were used relating both to the diagnostic confirmation of OSCC and to the immunohistochemical and molecular evaluation of HPV status. Additionally, to reduce the tissue alteration associated with the fixation, inclusion and microtomy procedures for the samples, the use of fresh tissue for molecular evaluation by PCR was preferred.

Using these selective and procedural criteria, the HPV frequency in the site-coded OSCCs was found to be low (4/40; 10%). All isolated cases were associated with high-risk genotypes (31, 51, 66, 67, 68), and, in two OSCC cases, the HPV status of the p16 IHC (negative) and PCR (positive) results did not match.

To the best of our knowledge, this is the first frequency study using a combination of infection diagnostic techniques (p16 IHC and PCR HPV-DNA) and applying the latest site/histological-coding systems to a sample of OSCCs, according to the most recent academic indications (2021 NIH/SEER ICD-0-3.2).

Thereafter, and to compare these findings with similar data in the literature, the relevant studies were critically reviewed, investigating HPV status using p16 and PCR from SCC in strictly oral sites. To maximize the accuracy of the data comparison, a re-coding of the oral sites was performed for each study by applying the same site-coding models used by the authors of this study.

Of the 13 selected studies, the overall HPV frequency, according to the PCR HPV-DNA detection method, ranged from 0% to 44%. However, this range was drastically reduced (from 3.3 to 12.5%) when only considering the three studies that correctly distinguished the ‘anterior 2/3 of tongue (C02.3) from the generic ‘tongue, NOS (C02.9)’. Only nine studies produced data for both HPV techniques [14,16,17,18,19,21,22,25,26], and in only one study did the HPV status of the p16 IHC and PCR results match (7/81 by both methods) [21]. Of the remaining eight studies, the number of p16 IHC positive results in HPV PCR DNA positive cases was observed to be discordant, with a sensitivity and specificity, between p16 IHC and PCR DNA, ranging from 0 to 65% and from 52 to 98%, respectively. Moreover, it was possible to detect a percentage of false positives (p16 IHC positives in HPV PCR DNA negative cases) in all nine studies, with a frequency ranging from 0% to 48%.

Therefore, both the results described in the observational study and those relating to the critical review confirmed the risk of an overestimation of HPV positivity in all OSCCs that had not been suitably distinguished by site, particularly relating to the tongue, and the frequent false HPV status using only p16 as a detection technique.

As suggested by several authors [29,30], especially regarding the oral epithelium, the expression of p16 is more variable than in the oropharynx [29,30]. It can, therefore, be less robust when used as an indirect biomarker for the transcriptional activity of hrHPV in the oral cavity [31]. Continuing to apply, also for oral cavity, the recommended guidelines regarding the detection of HPV in oropharyngeal squamous cell carcinomas (i.e., primary p16 IHC followed, only if positive, by PCR or in situ hybridization/ISH for HPV DNA), an unknown but perhaps higher number of OSCCs will very probably continue to be underdiagnosed, according to the related HPV status. Consequently, as HPV status is required to make treatment decisions to either de-escalate treatment or to apply new molecularly targeted therapies (using the p16 alone for viral detection, also regarding the oral cavity), a considerable number of patients could receive clinically inappropriate treatment [28,32].

## 5. Conclusions

In conclusion, within the limit of the sample size, the authors of this study contend that the results of the observational pilot study can support the skeptical interpretation of the role of HPV infection in oral carcinogenesis. This suggestion is further supported by a critical review of comparable research. Further investigations are required, necessarily conducted with updated and standardized site-coding systems (strictly excluding carcinomas at the base of the tongue) relating to OSCCs and with a more discriminative algorithm relating to the identification of viral transcriptional activity.

## Figures and Tables

**Table 1:** Information regarding sex, family history of cancer, tobacco (packs/year) and alcohol consumption (drink units-DU/week), and mechanical trauma.

	OSCC (No. 40)	
	N	(%)
**Sex**		
Female	17	(42.5)
Male	23	(57.5)
**Family history of cancer**		
Negative	40	(100)
Positive	0	(0)
**Tobacco consumption (packs/year)**		
Non-smokers (0)	19	(47.5)
Light-smokers (<25)	3	(7.5)
Moderate/heavy-smokers (>25)	18	(45)
**Alcohol consumption (DU/week)**		
Non-drinkers (0 or 1)	37	(92.5)
Moderate-drinkers (<16)	0	(0)
Heavy-drinkers (>16)	3	(7.5)
**Mechanical trauma**		
Sharp cusps	12	(30)
Incongruous prosthesis	3	(7.5)

**Table 2:** HPV frequency in site-coded OSCC.

OSCC Site (By New NIH/SEER ICD-0-3.2 System)	No. (%, 95% CI)	No. HPV-Positive OSCC (%, 95% CI)	Sex/Age	PCR HPV-DNA	p16 IHC
Border of tongue (C02.1)	13 (32.5%, 95% CI = [19–49%])	2 (15%, 95% CI = [2–45%])	M/65	HR-HPV 31 + 68	−
			M/65	HR-HPV 66	−
Overlapping lesions of tongue/no base of tongue (C02.8 = C02.1 + C02.2)	4 (10%, 95% CI = [3–24%])	0	−	−	−
Gum (C03.1)	2 (5%, 95% CI = [0.6–17%])	0	−	−	−
Anterior floor of mouth (C04.0)	4 (10%, 95% CI = [3–24%])	1 (25%, 95% CI = [0.6–80%])	M/44	HR-HPV 51	+
Hard palate (C05.0)	3 (7.5%, 95% CI = [2–20%])	0	−	−	−
Buccal mucosa (C06.0)	7 (17.5%, 95% CI = [7–33%])	0	−	−	−
Retromolar area (C06.2)	7 (17.5%, 95% CI = [7–33%])	1 (14%, 95% CI = [0.3–58%])	M/61	HR-HPV 67	+

Site = site-coded OSCC by 2021 NIH/SEER ICD-0-3.2 classification code system. Sex = M (male); Age = age (years) at the moment of diagnosis; PCR DNA= polymerase chain reaction DNA; p16 IHC = p16 immunohistochemistry; PCR DNA/p16IHC results= + (positive), − (negative).

**Table 3:** Detailed PCR HPV-DNA and p16 IHC results in 40 OSCCs.

HPV Test Results	No./Total OSCC (%, 95% CI)	HPV-Positive OSCC Sites (by 2021 NIH/SEER ICD-0-3.2 System)
PCR DNA (+)	4/40 (10%, 95% CI = [2–24%])	Retromolar area (C06.2) Anterior floor of mouth (C04.0) n.2 Border of the tongue (C02.1)
PCR DNA (−)	36/40 (90%, 95% CI = [76–97%])	—
p16 IHC (+)	2/40 (5%, 95% CI = [0.6–17%])	Retromolar area (C06.2) Anterior floor of mouth (C04.0)
p16 IHC (−)	38/40 (95%, 95% CI = [83–99%])	—
PCR DNA (+) p16 IHC (+)	2/40 (5%, 95% CI = [0.6–17%])	Retromolar area (C06.2) Anterior floor of mouth (C04.0)
PCR DNA (−) p16 IHC (−)	36/40 (90%, 95% CI = [76–97%])	—
PCR DNA (+) p16 IHC (−)	2/40 (5%, 95% CI = [0.6–17%])	n.2 Border of the tongue (C02.1)
PCR DNA (−) p16 IHC (+)	0/40 (0%, 95% CI = [0–9%])	—

**Table 4:** Summary of HPV frequency studies from 2010 to 2020, using PCR and p16 as viral identification techniques and re-coding by the 2021 NIH/SEER ICD-0-3.2 classification of OSCC sites. In bold are the three studies that reported a distinction between the ‘anterior 2/3 of tongue/C02.3’ and the generic ‘tongue, NOS (C02.9)’, with the following HPV frequency rates.

Authors (Year)	No.OSCCs Cases (Tissue Sample)	No. HPV-DNA PCR Positive Cases (%, 95% CI)	2021 NIH/SEER ICD-0-3.2 Site-Coded HPV-DNA PCR Positive Cases	No. p16 Positive Cases/No. HPV-DNA PCR Positive Cases (Sensitivity %, 95% CI)	No. p16 Negative/No. HPV-DNA PCR Positive Cases (Specificity%, 95% CI)	No. p16 Positive Cases/No. HPV-DNA PCR Negative Cases (%, 95% CI)
Harris et al. (2010) [14]	25 (PE)	2/25 (8%, 95% CI = [0.9–26%])	2 tongue, NOS (C02.9)	0/2 (0%, 95% CI = [0–84%])	12/23 (52%, 95% CI = [31–73%])	11/23 (48%, 95% CI = [27–69%])
**Laco et al. (2011) [15]**	**24 (FFPE)**	**3/24 (12,5%, 95% CI = [2–32%])**	**1 cheek mucosa (C06.0)** **1 gum, NOS (C03.9)** **1 anterior 2/3 of tongue, NOS (C02.3)**	**not reported**	**not reported**	**not reported**
Elango et al. (2011) [20]	60 (PE)	29/60 (48%, 95% CI = [35–62%])	29 tongue, NOS (C02.9)	not reported	not reported	not reported
Duray et al. (2012) [19]	147 (FFPE)	65/147 (44%, 95% CI = [36–53%])	13 tongue, NOS (C02.9) 12 floor of the mouth, NOS (C04.9) 2 gum, NOS (C03.9) 1 cheek mucosa (C06.0)	42/65 (65%, 95% CI = [52–76%])	46/82 (56%, 95% CI = [45–67%])	36/82 (44%, 95% CI = [33-55%])
Duncan et al. (2013) [21]	81 (FFPE)	7/81 (8.6%, 95% CI = [3–17%])	13 tongue, NOS (C02.9) 2 gum, NOS (C03.9) 12 floor of the mouth, NOS (C04.9) 1 cheek mucosa (C06.0) 1 retromolar area (C06.2) 2 lip, NOS (C00.9) 1 palate, NOS (C05.9) 33 mouth, NOS (C06.9)	7/7 (100%, 95% CI = [59–100%])	67/74 (91%, 95% CI = [81–96%]	7/74 (9,4%, 95% CI = [4–19%])
Ndiaye et al. (2013) [22]	41 (PE)	1/41 (2.4%, 95% CI = [0.06–13%])	1 gum, NOS (C03.9)	0/1 (0%, 95% CI = [0–98%])	40/40 (100%, 95% CI = [91–100%])	0/40 (0%, 95% CI = [0–88%])
Rushatamukayanunt et al. (2014) [23]	275 (FFPE)	69/275 (25.1%, 95% CI = [20–31%])	29 floor of the mouth, NOS (C04.9) 16 tongue, NOS (C02.9) 2 upper gum (C03.0) 13 lower gum (C03.1) 4 cheek mucosa (C06.0) 5 lip, NOS (C00.9)	not reported	not reported	not reported
Kruger et Al (2014) [24]	88 (FF)	5/88 (6%, 95% CI = [1.9–13%])	2 tongue, NOS (C02.9) 1 upper gum (C03.0) 1 lower gum (C03.1) 1 cheek mucosa (C06.0)	not reported	not reported	not reported
Singh et al. (2015) [25]	250 (FFPE)	23/250 (9.2%, 95% CI = [6–13%])	12 cheek mucosa (C06.0) 4 lower gum (C03.1) 1 retromolar area (C06.2) 6 tongue, NOS (C02.9)	9/23 (39.1%, 95% CI = [20–61%])	220/227 (97%, 95% CI = [94–99%])	7/227 (3.1%, 95% CI = [1–6%])
Reyes et al. (2015) [26]	80 (FFPE)	9/80 (11%, 95% CI = [5–20%])	3 tongue, NOS (C02.9)	2/9 (22%, 95% CI = [3–60%])	not reported	not reported
Blumberg et al. (2015) [16]	29 (FFPE)	0 (0%)	---	0	27/29 (93%, 95% CI = [77–99%])	2/29 (6.9%, 95% CI = [0.8–23%])
**Emmett et al. (2017) [17]**	**63 (FFPE)**	**5/63 (8%, 95% CI = [3–18%])**	**4 tongue (excluding base of tongue), NOS (C02.9)** **1 floor of the mouth, NOS (C04.9)**	**1/5 (20%, 95% CI = [0.5–72%])**	**57/58 (98%, 95% CI = [91–100%])**	**1/58 (1.7%, 95% CI = [0.04–9%])**
**Vidal Loustao et al. (2019) [18]**	**152 (FFPE)**	**5/152 (3.3%, 95% CI = [1–7%])**	* floor of the mouth, NOS (C04.9) * anterior 2/3 of tongue, NOS (C02.3)	**1/5 (20%, 95% CI = [0.5–72%])**	**136/147 (93%, 95% CI = [87–96%])**	**11/147 (7.5%, 95% CI = [4–13%])**

* Proportion not reported. FF = formalin-fixed; PE = paraffin embedded; FFPE = formalin fixed/paraffin embedded.

## Data Availability

Data available on request.

## References

[1] Fakhry C., Lacchetti C., Rooper L.M., Jordan R.C., Rischin D., Sturgis E.M., Bell D., Lingen M.W., Harichand-Herdt S., Thibo J. (2018). Human papillomavirus testing in head and neck carcinomas: ASCO clinical practice guideline endorsement of the college of American pathologists guideline. J. Clin. Oncol..

[2] Schache A.G., Liloglou T., Risk J.M., Filia A., Jones T.M., Sheard J., Woolgar J.A., Helliwell T.R., Triantafyllou A., Robinson M. (2011). Evaluation of human papilloma virus diagnostic testing in oropharyngeal squamous cell carcinoma: Sensitivity, specificity, and prognostic discrimination. Clin. Cancer Res..

[3] Bishop J.A., Ma X.J., Wang H., Luo Y., Illei P.B., Begum S., Taube J.M., Koch W.M., Westra W.H. (2012). Detection of transcriptionally active high-risk HPV in patients with head and neck squamous cell carcinoma as visualized by a novel E6/E7 mRNA in situ hybridization method. Am. J. Surg. Pathol..

[4] Campisi G., Panzarella V., Warnakulasuriya S., Greenspan J.S. (2020). Human Papillomavirus Infection: A Risk Factor for Oral and Oropharyngeal Cancers. Textbook of Oral Cancer: Prevention, Diagnosis and Management.

[5] Lydiatt W.M., Patel S.G., O’Sullivan B., Brandwein M.S., Ridge J.A., Migliacci J.C., Loomis A.M., Shah J.P. (2017). Head and neck cancers-major changes in the American Joint Committee on cancer eighth edition cancer staging manual. CA Cancer J. Clin..

[6] Mirghani H., Casiraghi O., Amen F., He M., Ma X.J., Saulnier P., Lacroix L., Drusch F., Lakdhar A.B., Saint Guily J.L. (2015). Diagnosis of HPV-driven head and neck cancer with a single test in routine clinical practice. Mod. Pathol..

[7] Syrjänen S. (2018). Oral manifestations of human papillomavirus infections. Eur. J. Oral Sci..

[8] World Health Organization (2013). International Classification of Diseases for Oncology.

[9] Ndiaye C., Mena M., Alemany L., Arbyn M., Castellsagué X., Laporte L., Bosch F.X., de Sanjosé S., Trottier H. (2014). HPV DNA, E6/E7 mRNA, and p16INK4a detection in head and neck cancers: A systematic review and meta-analysis. Lancet Oncol..

[10] National Cancer Institute (2020). Surveillance, Epidemiology, and E.R.P.-(NIH/SEER). Head and Neck Equivalent Terms and Definitions C000-C148, C300-C339, C410, C411, C442, C479 Excludes lymphoma and leukemia M9590–M9992 and Kaposi sarcoma M9140. https://seer.cancer.gov/tools/solidtumor/Head_Neck_STM.pdf.

[11] Donà M.G., Spriano G., Pichi B., Rollo F., Laquintana V., Covello R., Pellini R., Giuliani M., Pescarmona E., Benevolo M. (2015). Human papillomavirus infection and p16 overexpression in oropharyngeal squamous cell carcinoma: A case series from 2010 to 2014. Future Microbiol..

[12] Robinson M. (2017). HPV testing of head and neck cancer in clinical practice. Recent Results Cancer Res..

[13] Wasylyk B., Abecassis J., Jung A.C. (2013). Identification of clinically relevant HPV-related HNSCC: In p16 should we trust?. Oral Oncol..

[14] Harris S.L., Thorne L.B., Seaman W.T., Neil Hayes D., Couch M.E., Kimple R.J. (2011). Association of p16INK4a overexpression with improved outcomes in young patients with squamous cell cancers of the oral tongue. Head Neck.

[15] Laco J., Vosmikova H., Novakova V., Celakovsky P., Dolezalova H., Tucek L., Nekvindova J., Vosmik M., Cermakova E., Ryska A. (2011). The role of high-risk human papillomavirus infection in oral and oropharyngeal squamous cell carcinoma in non-smoking and non-drinking patients: A clinicopathological and molecular study of 46 cases. Virchows Arch..

[16] Blumberg J., Monjane L., Prasad M., Carrilho C., Judson B.L. (2015). Investigation of the presence of HPV related oropharyngeal and oral tongue squamous cell carcinoma in Mozambique. Cancer Epidemiol..

[17] Emmett S., Jenkins G., Boros S., Whiteman D.C., Panizza B., Antonsson A. (2017). Low prevalence of human papillomavirus in oral cavity squamous cell carcinoma in Queensland, Australia. ANZ J. Surg..

[18] Vidal Loustau A.C., Dulguerov N., Curvoisier D., McKee T., Lombardi T. (2019). Low prevalence of HPV-induced oral squamous cell carcinoma in Geneva, Switzerland. Oral Dis..

[19] Duray A., Descamps G., Decaestecker C., Remmelink M., Sirtaine N., Lechien J., Ernoux-Neufcoeur P., Bletard N., Somja J., Depuydt C.E. (2012). Human papillomavirus DNA strongly correlates with a poorer prognosis in oral cavity carcinoma. Laryngoscope.

[20] Elango K.J., Suresh A., Erode E.M., Subhadradevi L., Ravindran H.K., Iyer S.K., Iyer S.K.R., Kuriakose M.A. (2011). Role of human papilloma virus in oral tongue squamous cell carcinoma. Asian Pac. J. Cancer Prev..

[21] Duncan L.D., Winkler M., Carlson E.R., Heidel R.E., Kang E., Webb D. (2013). P16 immunohistochemistry can be used to detect human papillomavirus in oral cavity squamous cell carcinoma. J. Oral Maxillofac. Surg..

[22] Ndiaye C., Alemany L., Diop Y., Ndiaye N., Diémé M.J., Tous S., Klaustermeier J.E., Alejo M., Castellsagué X., Bosch F.X. (2013). The role of human papillomavirus in head and neck cancer in Senegal. Infect. Agent. Cancer.

[23] Rushatamukayanunt P., Morita K.I., Matsukawa S., Harada H., Shimamoto H., Tomioka H., Omura K. (2014). Lack of association between high-risk human papillomaviruses and oral squamous cell carcinoma in young Japanese patients. Asian Pac. J. Cancer Prev..

[24] Krüger M., Pabst A.M., Walter C., Sagheb K., Günther C., Blatt S., Weise K., Al-Nawas B., Ziebart T. (2014). The prevalence of human papilloma virus (HPV) infections in oral squamous cell carcinomas: A retrospective analysis of 88 patients and literature overview. J. Cranio Maxillofac. Surg..

[25] Singh V., Husain N., Akhtar N., Kumar V., Tewari S., Mishra S., Misra S., Khan M.Y. (2015). Do human papilloma viruses play any role in oral squamous cell carcinoma in North Indians. Asian Pac. J. Cancer Prev..

[26] Reyes M., Rojas-Alcayaga G., Pennacchiotti G., Carrillo D., Muñoz J.P., Peña N., Montes R., Lobos N., Aguayo F. (2015). Human papillomavirus infection in oral squamous cell carcinomas from Chilean patients. Exp. Mol. Pathol..

[27] Campisi G., Giovannelli L., Calvino F., Matranga D., Colella G., Di Liberto C., Capra G., Leao J.C., Lo Muzio L., Capogreco M. (2006). HPV infection in relation to OSCC histological grading and TNM stage. Evaluation by traditional statistics and fuzzy logic model. Oral Oncol..

[28] Qureishi A., Mawby T., Fraser L., Shah K.A., Møller H., Winter S. (2017). Current and future techniques for human papilloma virus (HPV) testing in oropharyngeal squamous cell carcinoma. Eur. Arch. Oto Rhino Laryngol..

[29] Bradley K.T., Budnick S.D., Logani S. (2006). Immunohistochemical detection of p16INK4a in dysplastic lesions of the oral cavity. Mod. Pathol..

[30] Chung C.H., Zhang Q., Kong C.S., Harris J., Fertig E.J., Harari P.M., Wang D., Redmond K.P., Shenouda G., Trotti A. (2014). p16 protein expression and human papillomavirus status as prognostic biomarkers of nonoropharyngeal head and neck squamous cell carcinoma. J. Clin. Oncol..

[31] Hendawi N., Niklander S., Allsobrook O., Khurram S.A., Bolt R., Doorbar J., Speight P.M., Hunter K.D. (2020). Human papillomavirus (HPV) can establish productive infection in dysplastic oral mucosa, but HPV status is poorly predicted by histological features and p16 expression. Histopathology.

[32] Taberna M., Mena M., Tous S., Pavón M.A., Oliva M., León X., Garcia J., Guix M., Hijano R., Bonfill T. (2018). HPV-relatedness definitions for classifying HPV-related oropharyngeal cancer patient do impact on TNM classification and patients’ survival. PLoS ONE.

